# Variables in the ACBD5 Gene Leading to Distinct Phenotypes: A Case Report

**DOI:** 10.7759/cureus.32930

**Published:** 2022-12-25

**Authors:** Mariella C Pappaterra-Rodriguez, Sofia M Muns, Sofía C Ayala Rodríguez, Guillermo A Requejo Figueroa, Natalio Izquierdo, Armando L Oliver

**Affiliations:** 1 School of Medicine, Ponce Health Sciences University, Ponce, PRI; 2 Ophthalmology, University of Puerto Rico School of Medicine, Medical Sciences Campus, San Juan, PRI; 3 Surgery, University of Puerto Rico School of Medicine, Medical Sciences Campus, San Juan, PRI

**Keywords:** case report, acbd5 variant, variants of uncertain significance, rod-cone dystrophy, inherited retinal disorders

## Abstract

We report the cases of a father and his daughter, the former diagnosed with retinitis pigmentosa (RP) and the latter with early foveal atrophy; while both shared a novel variant of uncertain significance (VUS) in the *ACBD5* gene (variant c.431G>A), they exhibited different clinical profiles and disease manifestations. The father was a 48-year-old man who presented with nyctalopia that had persisted since age seven. He had mild disk pallor, vessel attenuation, retinal pigment epithelium (RPE) changes nasal to the fovea, and few mid-peripheral bone spicules. Sequencing analysis showed that he carried seven VUS in five genes: *ACBD5 *c.431G>A (p.Gly144Asp), *CYP4V2* c.296T>C (p.Met99Thr), *EYS* c.1852G>A (p.Gly618Ser), *HMCN1 *c.280G>A (p.Val94Met), *HMCN1 *c.8939A>C (p.Asn2980Thr), *RP1L1 *c.575C>A (p.Pro192His), and *RP1L1 *c.1375A>C (p.Thr459Pro). He shared only the *ACBD5* gene with his 18-year-old daughter. The daughter had 20/20 visual acuity, but further testing showed foveal atrophy and hyperautofluorescence. Intrafamilial phenotypic heterogeneity was detected in our patients. Studies on the role of hormonal factors leading to phenotypic variability are warranted.

## Introduction

Retinitis pigmentosa (RP) is a heterogeneous group of inherited retinal dystrophies characterized by the progressive loss of photoreceptors and the presence of retinal pigment deposits on fundus examination [1,2]. The most prevalent form of RP is rod-cone dystrophy, which manifests with nyctalopia, followed by loss of peripheral vision, then central vision, finally leading to legal blindness [1].

According to Hartong et al., the worldwide prevalence of RP is 1:4000 [3]. RP may be inherited as an autosomal dominant, autosomal recessive, or X-linked trait. Cases may occur sporadically [4]. There are multiple genes associated with this disease, including genes inherited as autosomal dominant, such as *RP1*, autosomal recessive, such as *CYP4V2* and *EYS*, and X-linked, such as *RP2* and *RPGR *[5]. However, RP has been associated with a high mutational load and many of the genes associated with these diseases have not been described yet [1,4].

The genetic complexity of RP inheritance could partially explain the phenotypic heterogeneity among RP patients. However, it has been recently suggested that phenotypic differences among patients with retinal diseases might reflect the role of environmental influences, such as hormonal variations [6-8]. Therefore, the mechanisms behind the phenotypic spectrum among patients with retinal diseases, such as RP, are not yet fully understood.

In this report, we present the cases of a father and his daughter. The former carried a diagnosis of RP and the latter had early foveal atrophy. Both shared a variant of uncertain significance (VUS) in the *ACBD5* gene (variant c.431G>A), yet exhibited different clinical profiles and disease manifestations.

## Case presentation

Patient 1

A 48-year-old male patient presented with complaints of nyctalopia and peripheral vision loss. His grandparents were cousins, indicating consanguinity in the family. His mother had glaucoma and his daughter had foveal hyperautofluorescence and foveal atrophy (described below as Patient 2).

**Figure 1:**
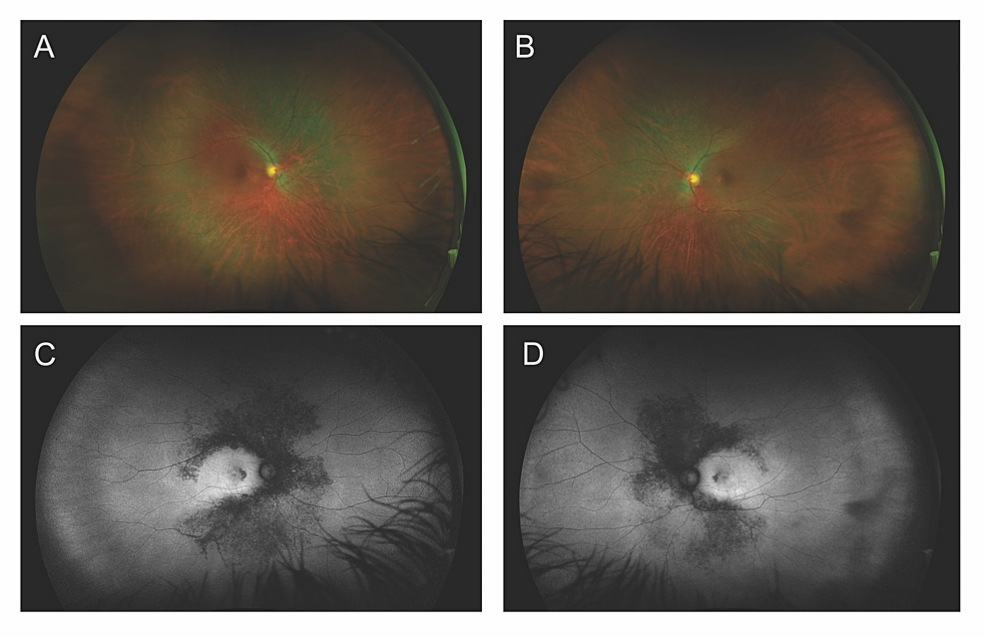
Ultra-widefield fundus images of Patient 1 Color photography of the right (A) and left (B) eyes show mild disk pallor, vessel attenuation, retinal pigment epithelium (RPE) changes nasal to the fovea, and few mid-peripheral bone spicules. Fundus autofluorescence of the right (C) and left (D) eyes show decreased autofluorescence in the mid-periphery, corresponding with the previously noted RPE changes; the macula, on the other hand, shows increased autofluorescence

Upon a comprehensive ophthalmic examination, he had a best-corrected visual acuity of 20/25 in both eyes (OU). Intraocular pressures were 14 mmHg OU, and Ishihara color plates were 2/14 and 4/14, in the right (OD) and left eye (OS), respectively. Anterior segment slit-lamp examination was unremarkable. As depicted in Figures 1A, 1B, upon indirect ophthalmoscopy, the patient had mild disk pallor, vessel attenuation, retinal pigment epithelium (RPE) changes nasal to the fovea, and few mid-peripheral bone spicules. Additionally, decreased autofluorescence in the mid-periphery was noted on ultra-widefield fundus autofluorescence imaging. On the other hand, as shown in Figures 1C, 1D, increased autofluorescence was observed in the macula.

**Figure 2:**
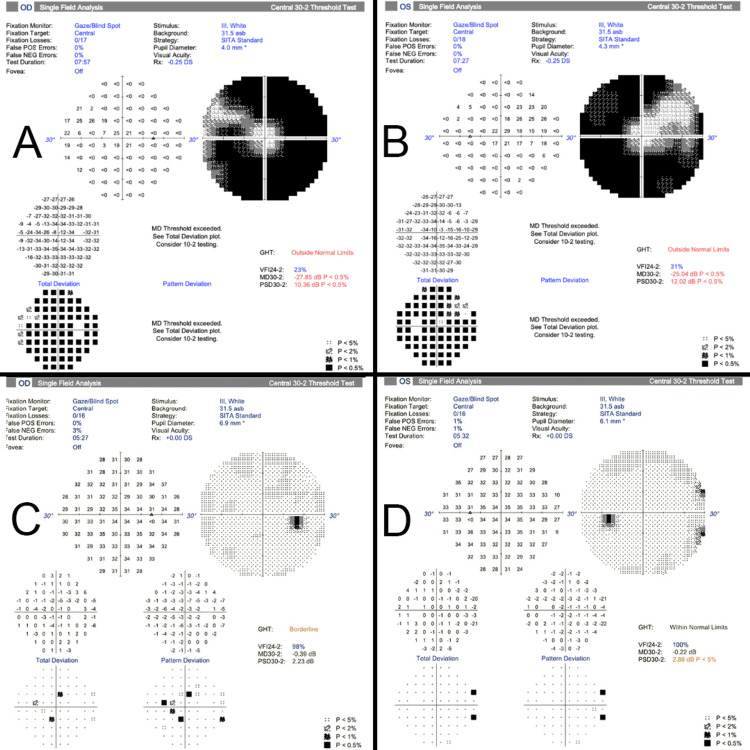
Humphrey visual field testing (central 30-2 threshold test, stimulus III, white, SITA-Standard) Right (A) and left (B) visual fields of Patient 1 show severe peripheral constriction with central and superonasal sparing, while Patient 2's visual fields show a central depression in the right eye (C) and a borderline-high pattern standard deviation value in the left eye (D) SITA: Swedish Interactive Thresholding Algorithm

Upon Humphrey visual field examination (central 30-2 threshold test), our patient had severe peripheral constriction with central and superonasal sparing OU, as illustrated in Figures 2A, 2B. Full-field electroretinogram (ERG) showed markedly decreased a- and b-wave amplitudes in the dark- and light-adapted responses to bright flashes, severely decreased scotopic b-wave amplitude, and delayed flicker responses, bilaterally (dark-adapted 0.01 ERG; dark-adapted 3.0 ERG; dark-adapted 30.0 ERG; dark-adapted 3.0 OPs; light-adapted 3.0 ERG; light-adapted 3.0 flicker ERG).

Further work-up, including rapid plasma reagin and fluorescent treponemal antibody test, was negative. However, his serum vitamin A levels were 63.2 ug/dL, which was above the upper limit of the reference interval (62.0 ug/dL). Molecular Vision Laboratory (Hillsboro, OR) results showed heterozygous VUS mutations on the *RP1L1* gene. Variants found on the *RP1L1 *gene were c.1375A>C (p.Thr459Pro) and c.575C>A (p.Pro192His). These variants were not found in the gnomAD database. 

Upon next-generation sequencing using an Invitae IRD panel (Invitae Corporation, San Francisco, CA), three heterozygous VUS were detected for this patient: *ACBD5* gene [variant c.431G>A (p.Gly144Asp)]; *CYP4V2* gene [variant c.296T>C (p.Met99Thr)]; and *EYS* gene [variant c.1852G>A (p.Gly618Ser)]. 

Patient 2

**Figure 3:**
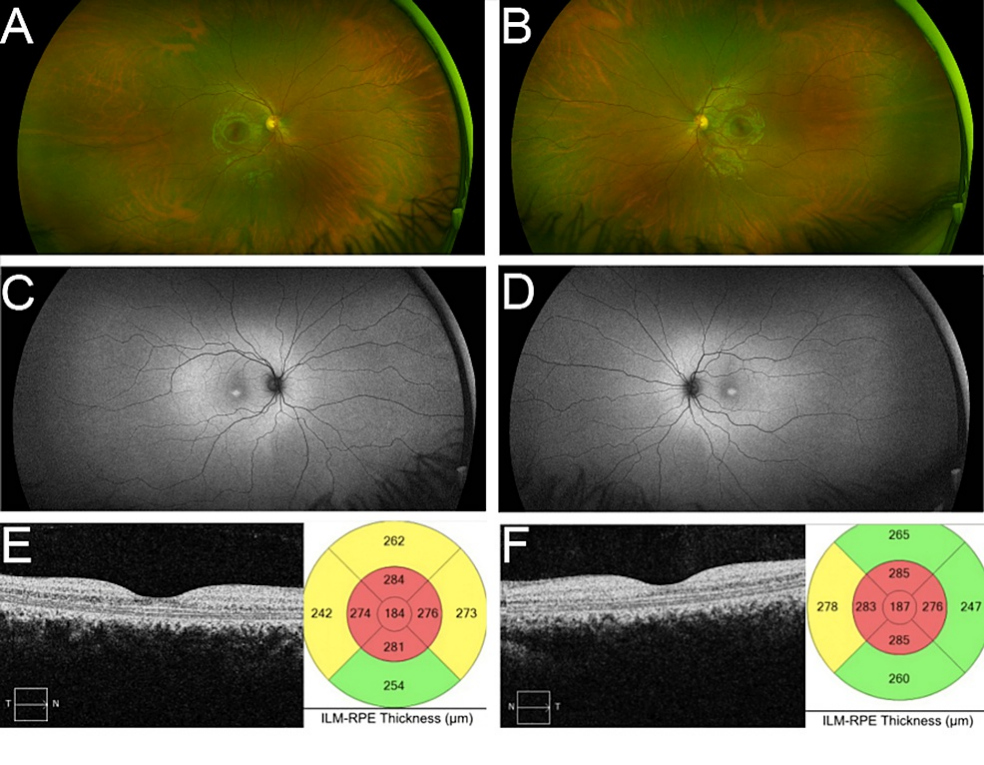
Ultra-widefield fundus images of Patient 2 Color photography of the right (A) and left (B) eyes shows a seemingly normal fundus. However, fundus autofluorescence shows foveal hyperautofluorescence with surrounding hypoautofluorescence in the right (C) and left (D) maculae. Spectral-domain optical coherence tomography shows significant right (E) and left (F) foveal atrophy

An 18-year-old female patient, who carried a foveal atrophy diagnosis, returned for her ophthalmic evaluation. Upon a comprehensive ophthalmic evaluation, the patient had an uncorrected visual acuity of 20/20 in both eyes, normal color vision (tested by Ishihara plates), and an unremarkable anterior segment examination. Fundus examination seemed unremarkable, as shown in Figures 3A, 3B. On the other hand, fundus autofluorescence showed foveal hyperautofluorescence surrounded by mild hypoautofluorescence, as depicted in Figures 3C, 3D. Spectral-domain OCT showed significant foveal atrophy OU, as illustrated in Figures 3E, 3F.

**Figure 4:**
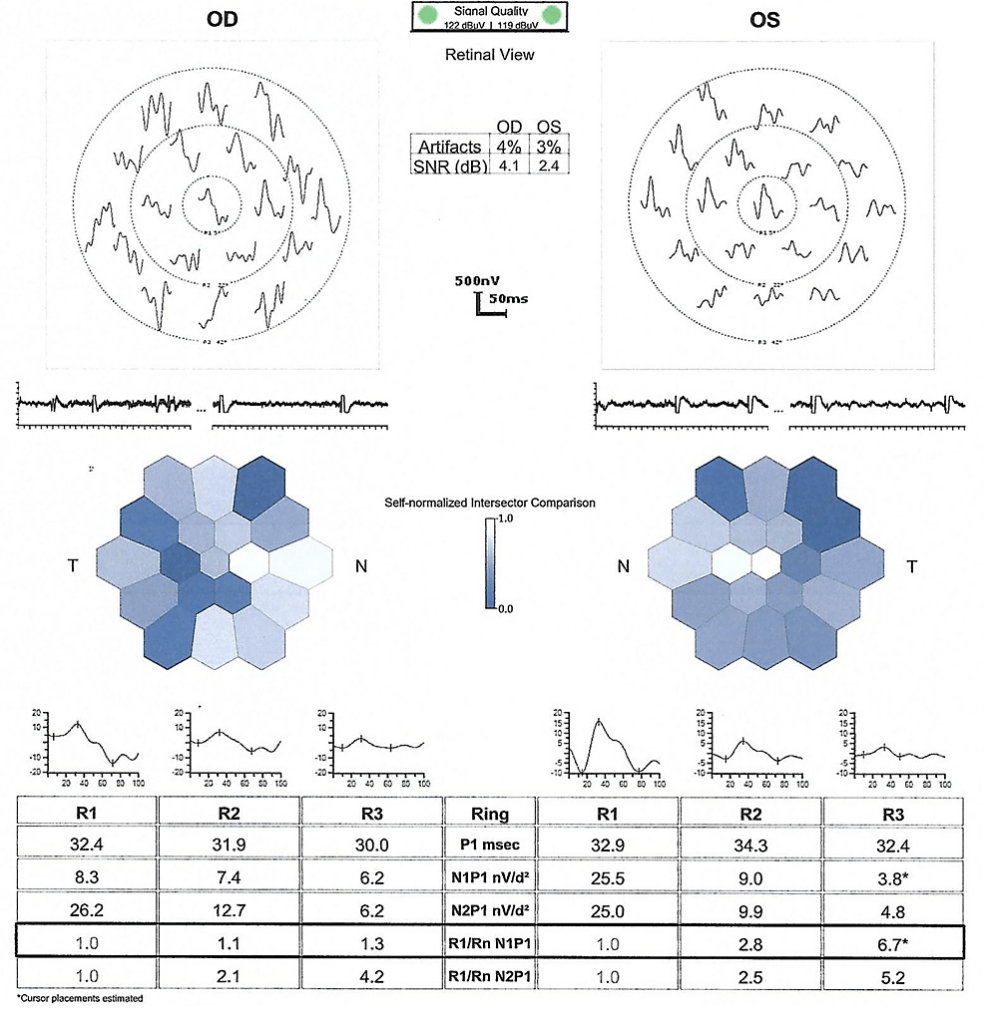
Multifocal ERGs of Patient 2 Multifocal ERG test (Diopsys® mfERG) shows abnormal waveforms in both eyes. P1 amplitudes ranged from 30.0 to 32.4 and from 32.4 to 34.3 in the OD and OS, respectively ERG: electroretinogram

Upon Humphrey visual field examination (central 30-2 threshold test), this patient had a central depression and a high pattern standard deviation value, in the OD and OS, respectively, as depicted in Figures 2C, 2D. She had an unremarkable full-field ERG with an abnormal multifocal ERG as shown in Figure 4.

Genetic testing (Invitae Corporation, San Francisco, CA) results showed two heterozygous VUS on gene *ACBD5* [variant c.431G>A (p.Gly144Asp)] and on gene *NPHP3* [variant c.1027A>G (p.Ile343Val)]. The *ACBD5* missense mutation was the same one found in her father (Patient 1). In silico expression analysis was not performed.

## Discussion

Numerous genetic variants have been implicated in the phenotypic heterogeneity associated with RP, many of which have yet to be reported, as shown in Table 1 [4]. Algorithms that predict the effects of mutations have categorized the variants. The *ACBD5* c.431G>A variant was predicted to be both "tolerated" by some authors and “probably damaging” by others; neither prediction has so far proved to be accurate [8-11].

**Table 1:** Variants of uncertain significance and their associated inherited retinal dystrophies ^†^Present in the father and daughter who are the subjects of this report. ^††^Present in the father, one of the subjects of this report. ^‡^Present in the father, one of the subjects of this report

Variants	Inherited retinal dystrophy
*ACBD5* (p.Gly144Asp)^† ^	Not previously reported in the literature
*CYP4V2* (p.Met99Thr)^†† ^	Not previously reported in the literature
*EYS* (p.Gly618Ser)^‡ ^	Autosomal recessive retinitis pigmentosa (arRP)
*HMCN1* (p.Val94Met)^‡^	Not previously reported in the literature
*HMCN1* (p.Asn2980Thr)^‡^	Age-related macular degeneration 1
*NPHP3* (p.Ile343Val)	Reported in association with nephronophthisis
*RP1L1* (p.Thr459Pro)^‡^	Not previously reported in the literature
*RP1L1* (p.Pro192His)^‡^	Not previously reported in the literature

Genetic variants found in our patients, *ACBD5* c.431G>A, *CYP4V2* c.296T>C, *HMCN1* c.280G>A, and *HMCN1* c.8939A>C, have not been formerly associated with RP degeneration [9-13]. However, the specific variants *ACBD5* c.431G>A found among our patients have not yet been described in the literature.

Both the father and his daughter shared the same novel VUS in the *ACBD5* gene (c.431G>A). However, the two patients had different phenotypes. While his fundus appearance was consistent with rod-cone dystrophy (Figures 1A, 1B), his daughter had a normal-appearing fundus (Figures 3A, 3B), with subtle anomalies that became evident only on ancillary testing (Figures 3C, 3D, 3E, 3F). Most certainly, the daughter’s maculopathy will continue to worsen as she ages. Her full-field ERG was normal.

A limitation of this study is that the mfERG recording obtained to a stimulus array contained 19 elements. In addition, it is unknown whether the two variants are located in cis or trans of *ACBD5,* *CYP4V2*, *EYS*, *HMCN1*, and *RP1L1 *individually. Sanger sequence or segregation from the family members such as parents, offsprings, or siblings of the proband are warranted in order to locate these variants.

Several theories have been proposed to explain the effects of these VUSs and their associations with RP. Audo et al. have described a patient with a diagnosis of autosomal recessive RP who had both the *EYS* c.1852G>A (which was found in Patient 1) and the *EYS* c.1642C>T variants [14]. The *EYS* gene has also been seen in association with the *RP1L1* gene as a causative agent of RP [15]. Additionally, mutations in the *RP1L1 *gene lead to autosomal recessive RP (type 88); therefore, the father's clinical picture can be attributed to being a compound heterozygote [5]. The symptoms presented by the father may have derived from the interaction between the *ACBD5*, *EYS*, and *RP1L1 *variants that he carried according to genetic testing. This may also explain why the daughter did not present with such severe symptoms, as she carried only the *ACBD5* gene mutation. This suggests that the *ACBD5* variant might work alone, but in more subtle ways, as seen in the manifestations of our 18-year-old patient. The differences between the phenotypes of these patients may exemplify the complex interactions between VUSs in the phenotypic variability of IRDs. However, the role of these VUSs remains controversial due to their clinical unpredictability [16].

In addition to genetic influences, other researchers have suggested that the phenotypic variations reported among patients with retinal diseases might reflect the role of environmental influences in the manifestations of the disease [6-8,17]. There is an ongoing debate about the role of hormones in the phenotypic variability and progression of retinal diseases [7,8,17]. Receptors for estrogen, progesterone, and androgen (testosterone) have been found in several ocular locations, including the retina [7]. Recent studies have suggested that estrogen derivatives might have protective effects on the retina by protecting retinal photoreceptor neurons from glutamate-induced damage, exerting antioxidant neuroprotective effects, and modulating ocular blood flow due to their vasodilatory properties [7,8,18]. Also, the distribution of these sex steroid hormone receptors in the retina varies by age and sex, which could help explain the differences in the epidemiology of certain eye diseases [17]. Further studies should be conducted to elucidate the role of hormones and VUS in the phenotypic expression of RP.

## Conclusions

We reported the cases of a father and his daughter, the former diagnosed with RP and the latter with early foveal atrophy, who shared a novel VUS, c.431G>A in the *ACBD5* gene. Both patients had phenotypic variability. Additionally, mutations in the *RP1L1* gene lead to autosomal recessive RP (type 88); therefore, being a compound heterozygote can explain the father's clinical picture. These findings may be attributed to hormonal influences, the effect of additional VUS, or the two variants being located in cis or trans of the *ACBD5* gene. Sanger sequence or segregation from the family members such as offsprings or siblings of the proband is warranted to locate these variants.
